# Dichlorido{[2-(diphenyl­phosphino)phenyl­imino­meth­yl]ferrocene-κ^2^ *N*,*P*}palladium(II) dichloro­methane hemi­solvate

**DOI:** 10.1107/S1600536808039949

**Published:** 2008-12-03

**Authors:** Huanyu Liu, Dongsheng Shen

**Affiliations:** a College of Pharmacy, Guangdong Pharmaceutical University, Guangzhou 510006, People’s Republic of China

## Abstract

There are two independent Pd^II^ complex mol­ecules in the asymmetric unit of the title compound, [PdCl_2_{Fe(C_5_H_5_)(C_24_H_19_NP)}]·0.5CH_2_Cl_2_. One ferrocenyl ring of one complex mol­ecule is disordered over two sites with half-occupancy for each component. Both Pd^II^ cations adopt a distorted square-planar coordination geometry with a bidentate [2-(diphenyl­phosphino)phenyl­imino­meth­yl]ferrocene ligand and two chloride anions.

## Related literature

For general background see: Reddy *et al.* (2000 ▶, 2002 ▶); Catsoulacos *et al.* (2003 ▶); Weng *et al.* (2004 ▶); Koprowski *et al.* (2002 ▶); For a related structure, see: Doherty *et al.* (2002 ▶). For the synthesis, see: Gong *et al.* (2006 ▶); Zhang *et al.* (2006 ▶).
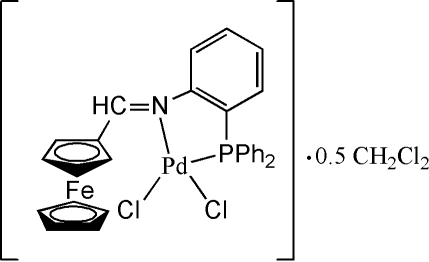


## Experimental

### 

#### Crystal data

[FePd(C_5_H_5_)Cl_2_(C_24_H_19_NP)]·0.5CH_2_Cl_2_
*M* *_r_* = 693.08
Triclinic, 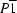

*a* = 11.7559 (4) Å
*b* = 11.8798 (4) Å
*c* = 21.8200 (7) Å
α = 76.9672 (4)°
β = 89.117 (1)°
γ = 70.3297 (4)°
*V* = 2789.51 (16) Å^3^
*Z* = 4
Mo *K*α radiation
μ = 1.53 mm^−1^
*T* = 293 (2) K
0.30 × 0.10 × 0.10 mm

#### Data collection

Bruker SMART CCD area-detector diffractometer
Absorption correction: multi-scan (*SADABS*; Sheldrick, 1996 ▶) *T*_min_ = 0.795, *T*_max_ = 0.868
21205 measured reflections
12145 independent reflections
9726 reflections with *I* > 2σ(*I*)
*R*_int_ = 0.018

#### Refinement

*R*[*F* ^2^ > 2σ(*F* ^2^)] = 0.029
*wR*(*F* ^2^) = 0.076
*S* = 1.04
12145 reflections
685 parameters
4 restraints
H-atom parameters constrained
Δρ_max_ = 0.99 e Å^−3^
Δρ_min_ = −1.04 e Å^−3^

### 

Data collection: *SMART* (Bruker, 1998 ▶); cell refinement: *SAINT* (Bruker, 1998 ▶); data reduction: *SAINT*; program(s) used to solve structure: *SHELXTL* (Sheldrick, 2008 ▶); program(s) used to refine structure: *SHELXTL*; molecular graphics: *SHELXTL*; software used to prepare material for publication: *SHELXTL*.

## Supplementary Material

Crystal structure: contains datablocks global, I. DOI: 10.1107/S1600536808039949/xu2460sup1.cif

Structure factors: contains datablocks I. DOI: 10.1107/S1600536808039949/xu2460Isup2.hkl

Additional supplementary materials:  crystallographic information; 3D view; checkCIF report

## Figures and Tables

**Table 1:** Selected bond lengths (Å)

Pd1—N1	2.051 (2)
Pd1—P1	2.2118 (6)
Pd1—Cl1	2.2832 (7)
Pd1—Cl2	2.3926 (6)
Pd2—N2	2.0616 (18)
Pd2—P2	2.1979 (6)
Pd2—Cl4	2.2875 (6)
Pd2—Cl3	2.3843 (7)

**Fig. 1.**
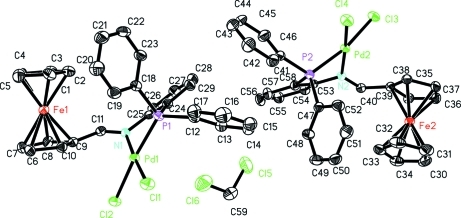
The molecular structure of the title compound. Displacement ellipsoids are drawn at 30% probability level, and all hydrogen atoms and the disorder component of C1'-C5' are omitted for clarity.

## supplementary crystallographic information

### Comment

Palladium(II) complexes with iminophosphine bidentate ligands have attracted much attention for their good catalysis such as the Heck reaction (Reddy *et al.*, 2000; Catsoulacos *et al.*, 2003), the Suzuki and Stille coupling reaction (Weng *et al.*, 2004; Koprowski *et al.*, 2002), the copolymerization of CO-ethylene (Reddy *et al.*, 2002). Many of alkanyl- or aryl- substituted iminophosphine ligands and their palladium(II) complexes have been reported, but related studies on ferrocenyl- substituted counterpart are still rare. Recently we synthesed a novel palladium(II) complex with ferrocenyliminophosphine ligand, its crystal structure is presented here.

There are two independent complex molecules in the asymmetric unit of the crystal structure of the compound (Fig. 1), and their molecular geometries are very similar. The Pd^II^ atoms adopt distorted square planar geometry, with P, N atoms from the bidentate ferrocenyliminophosphine ligand (*L*) and two Cl^-^ anions. The N1—Pt1—Cl1 and N2—Pt2—Cl4 bond angles are 174.86 (6)° and 171.66 (6)°, respectively. The ligand *L* adopts a five-membered chelating ring, and the benzene ring and the cyclopentadienyl ring in a *trans*-configuration about the C?N bond. The Pd—P, Pd—N and Pd—Cl bond distances (Table 1) are in the normal range (Doherty *et al.*, 2002). The average Pd—Cl bond length *trans* to the P atoms [Pd1—Cl2 and Pd2—Cl3] is longer than that *trans* to the N atoms [Pd1—Cl1 and Pd2—Cl4] owing to the stronger *trans* influence of a tertiary phosphine with respect to an imine.

### Experimental

Ligand *L* was prepared by literature method (Zhang *et al.*, 2006; Gong *et al.*, 2006). For the preparation of the compound, a suspension formed by anhydrous lithium chloride (58 mg, 0.135 mmol), anhydrous palladium chloride (120 mg, 0.676 mmol) and 5 ml of methanol was stirred at room temperature until a homogeneous solution was formed, then *L* (319 mg, 0.676 mmol) dissolved in 20 ml dichloromethane/methanol (1:1, V/V) was added and reacted for 20 h at room temperature. The obtained brown reaction mixture was concentrated in vacuum until a brown red solid formed. The crude browed product was washed for several times with a small amount of ether to afford a brown red solid (334 mg, yield 76%). Single crystals of the compound suitable for X-ray analysis were obtained by slow diffusion of petroleum ether into the CH_2_Cl_2_ solution of the compound. Analysis calculated for [PdCl_2_(C_29_H_24_FeNP].0.5(CH_2_Cl_2_): C 51.12, H 3.64, N 2.02%; Found: C 51.25, H 3.50, N 2.12%.

### Refinement

All H atoms were positioned geomertrically and treated as riding with C—H = 0.98 Å for cyclopentadienyl rings, C—H = 0.93 Å for benzene rings and C—H = 0.97 Å for methylene, and refined in riding mode with *U*_iso_(H) = 1.2*U*_eq_(C). One ferrocenyl ring (C1—C5) is disorder over two sites, and refined with 0.5 occupancy for each component. Restraint was used for atoms C1—C5 and C1'-C5' to enforce co-planar.

### Crystal data

**Table:**

[FePd(C_5_H_5_)Cl_2_(C_24_H_19_NP)]·0.5CH_2_Cl_2_	*Z* = 4
*M**_r_* = 693.08	*F*(000) = 1388
Triclinic, *P*1	*D*_x_ = 1.650 Mg m^−^^3^
Hall symbol: -P 1	Mo *K*α radiation, λ = 0.71073 Å
*a* = 11.7559 (4) Å	Cell parameters from 2018 reflections
*b* = 11.8798 (4) Å	θ = 3.1–28.1°
*c* = 21.8200 (7) Å	µ = 1.53 mm^−^^1^
α = 76.9672 (4)°	*T* = 293 K
β = 89.117 (1)°	Needle, brown yellow
γ = 70.3297 (4)°	0.30 × 0.10 × 0.10 mm
*V* = 2789.51 (16) Å^3^	

### Data collection

**Table:**

Bruker SMART CCD area-detector diffractometer	12145 independent reflections
Radiation source: fine-focus sealed tube	9726 reflections with *I* > 2σ(*I*)
graphite	*R*_int_ = 0.018
φ and ω scans	θ_max_ = 28.3°, θ_min_ = 1.8°
Absorption correction: multi-scan (*SADABS*; Sheldrick, 1996)	*h* = −15→14
*T*_min_ = 0.795, *T*_max_ = 0.868	*k* = −15→15
21205 measured reflections	*l* = −29→28

### Refinement

**Table:**

Refinement on *F*^2^	Primary atom site location: structure-invariant direct methods
Least-squares matrix: full	Secondary atom site location: difference Fourier map
*R*[*F*^2^ > 2σ(*F*^2^)] = 0.029	Hydrogen site location: inferred from neighbouring sites
*wR*(*F*^2^) = 0.076	H-atom parameters constrained
*S* = 1.04	*w* = 1/[σ^2^(*F*_o_^2^) + (0.04*P*)^2^ + 0.5*P*] where *P* = (*F*_o_^2^ + 2*F*_c_^2^)/3
12145 reflections	(Δ/σ)_max_ = 0.001
685 parameters	Δρ_max_ = 0.99 e Å^−^^3^
4 restraints	Δρ_min_ = −1.04 e Å^−^^3^

### Special details

**Table:**

Geometry. All e.s.d.'s (except the e.s.d. in the dihedral angle between two l.s. planes) are estimated using the full covariance matrix. The cell e.s.d.'s are taken into account individually in the estimation of e.s.d.'s in distances, angles and torsion angles; correlations between e.s.d.'s in cell parameters are only used when they are defined by crystal symmetry. An approximate (isotropic) treatment of cell e.s.d.'s is used for estimating e.s.d.'s involving l.s. planes.
Refinement. Refinement of *F*^2^ against ALL reflections. The weighted *R*-factor *wR* and goodness of fit *S* are based on *F*^2^, conventional *R*-factors *R* are based on *F*, with *F* set to zero for negative *F*^2^. The threshold expression of *F*^2^ > σ(*F*^2^) is used only for calculating *R*-factors(gt) *etc*. and is not relevant to the choice of reflections for refinement. *R*-factors based on *F*^2^ are statistically about twice as large as those based on *F*, and *R*- factors based on ALL data will be even larger.

### Fractional atomic coordinates and isotropic or equivalent isotropic displacement parameters (Å^2^)

**Table:**

	*x*	*y*	*z*	*U*_iso_*/*U*_eq_	Occ. (<1)
Pd1	0.273505 (16)	0.847407 (16)	0.044514 (8)	0.02967 (5)	
Fe1	0.20719 (4)	1.27230 (3)	0.031690 (18)	0.03787 (9)	
Cl1	0.14365 (7)	0.75836 (7)	0.01696 (3)	0.05167 (18)	
Cl2	0.34494 (6)	0.87645 (6)	−0.05914 (3)	0.04191 (15)	
P1	0.24403 (6)	0.80204 (6)	0.14615 (3)	0.03328 (14)	
N1	0.38446 (18)	0.92535 (17)	0.07718 (9)	0.0306 (4)	
C1	0.2739 (6)	1.3085 (12)	0.1096 (5)	0.062 (3)	0.50
H1A	0.3588	1.2961	0.1197	0.075*	0.50
C2	0.2150 (9)	1.2212 (5)	0.1297 (3)	0.056 (3)	0.50
H2A	0.2523	1.1377	0.1559	0.067*	0.50
C3	0.0930 (7)	1.2753 (11)	0.1045 (5)	0.061 (3)	0.50
H3A	0.0311	1.2362	0.1107	0.073*	0.50
C4	0.0765 (8)	1.3961 (10)	0.0688 (5)	0.063 (3)	0.50
H4A	0.0008	1.4555	0.0466	0.076*	0.50
C5	0.1883 (13)	1.4166 (6)	0.0720 (5)	0.055 (3)	0.50
H5A	0.2034	1.4925	0.0522	0.065*	0.50
C5'	0.2536 (9)	1.3732 (14)	0.0840 (5)	0.070 (3)	0.50
H5'A	0.3199	1.4061	0.0766	0.084*	0.50
C1'	0.2621 (12)	1.2561 (10)	0.1217 (5)	0.078 (4)	0.50
H1'A	0.3353	1.1941	0.1451	0.093*	0.50
C2'	0.1456 (18)	1.2452 (10)	0.1203 (5)	0.076 (5)	0.50
H2'A	0.1245	1.1737	0.1418	0.091*	0.50
C3'	0.0650 (6)	1.3556 (16)	0.0817 (6)	0.074 (4)	0.50
H3'A	−0.0210	1.3733	0.0714	0.089*	0.50
C4'	0.1318 (13)	1.4347 (6)	0.0592 (3)	0.057 (3)	0.50
H4'A	0.0997	1.5169	0.0311	0.068*	0.50
C6	0.1426 (3)	1.2649 (2)	−0.05426 (13)	0.0445 (6)	
H6A	0.0588	1.3051	−0.0720	0.053*	
C7	0.2345 (3)	1.3180 (2)	−0.06287 (13)	0.0456 (7)	
H7A	0.2251	1.4007	−0.0877	0.055*	
C8	0.3405 (3)	1.2341 (2)	−0.02750 (13)	0.0420 (6)	
H8A	0.4183	1.2467	−0.0249	0.050*	
C9	0.3152 (2)	1.1241 (2)	0.00204 (12)	0.0342 (5)	
C10	0.1902 (2)	1.1461 (2)	−0.01455 (12)	0.0377 (6)	
H10A	0.1462	1.0889	−0.0007	0.045*	
C11	0.3938 (2)	1.0313 (2)	0.05196 (12)	0.0334 (5)	
H11A	0.4574	1.0489	0.0677	0.040*	
C12	0.2152 (3)	0.6612 (2)	0.18022 (12)	0.0393 (6)	
C13	0.3090 (3)	0.5482 (3)	0.19411 (13)	0.0492 (7)	
H13A	0.3885	0.5447	0.1882	0.059*	
C14	0.2839 (4)	0.4410 (3)	0.21673 (15)	0.0605 (9)	
H14A	0.3466	0.3656	0.2265	0.073*	
C15	0.1672 (4)	0.4459 (3)	0.22469 (16)	0.0693 (10)	
H15A	0.1506	0.3736	0.2391	0.083*	
C16	0.0747 (4)	0.5557 (4)	0.21176 (18)	0.0742 (11)	
H16A	−0.0043	0.5577	0.2178	0.089*	
C17	0.0974 (3)	0.6653 (3)	0.18958 (15)	0.0548 (8)	
H17A	0.0340	0.7402	0.1812	0.066*	
C18	0.1299 (2)	0.9197 (2)	0.17543 (12)	0.0383 (6)	
C19	0.0207 (3)	0.9843 (3)	0.14081 (14)	0.0510 (7)	
H19A	0.0086	0.9713	0.1013	0.061*	
C20	−0.0708 (3)	1.0685 (3)	0.16482 (17)	0.0646 (9)	
H20A	−0.1448	1.1103	0.1419	0.078*	
C21	−0.0520 (3)	1.0901 (3)	0.22239 (16)	0.0622 (9)	
H21A	−0.1131	1.1472	0.2382	0.075*	
C22	0.0566 (3)	1.0277 (3)	0.25658 (14)	0.0590 (8)	
H22A	0.0692	1.0434	0.2953	0.071*	
C23	0.1475 (3)	0.9418 (3)	0.23391 (13)	0.0470 (7)	
H23A	0.2204	0.8986	0.2577	0.056*	
C24	0.3906 (2)	0.7893 (2)	0.17786 (12)	0.0344 (5)	
C25	0.4503 (2)	0.8555 (2)	0.13659 (11)	0.0326 (5)	
C26	0.5635 (2)	0.8549 (2)	0.15411 (13)	0.0394 (6)	
H26A	0.6031	0.8987	0.1265	0.047*	
C27	0.6162 (3)	0.7887 (3)	0.21282 (14)	0.0485 (7)	
H27A	0.6927	0.7866	0.2246	0.058*	
C28	0.5565 (3)	0.7245 (3)	0.25497 (14)	0.0498 (7)	
H28A	0.5927	0.6813	0.2949	0.060*	
C29	0.4451 (3)	0.7248 (2)	0.23787 (13)	0.0444 (6)	
H29A	0.4054	0.6821	0.2662	0.053*	
Pd2	0.671825 (16)	0.134965 (16)	0.483672 (8)	0.02981 (5)	
Fe2	0.88320 (3)	−0.23875 (3)	0.422838 (17)	0.03519 (9)	
Cl3	0.77691 (6)	0.04680 (7)	0.58505 (3)	0.04380 (16)	
Cl4	0.48418 (6)	0.18014 (7)	0.52275 (3)	0.04880 (17)	
P2	0.59242 (6)	0.25219 (6)	0.39076 (3)	0.03206 (14)	
N2	0.82794 (17)	0.09895 (17)	0.43592 (9)	0.0294 (4)	
C30	0.9100 (3)	−0.3508 (3)	0.36213 (16)	0.0605 (9)	
H30A	0.9684	−0.4346	0.3693	0.073*	
C31	0.7909 (4)	−0.3189 (4)	0.37920 (16)	0.0674 (10)	
H31A	0.7509	−0.3762	0.4001	0.081*	
C32	0.7377 (3)	−0.1902 (4)	0.36093 (15)	0.0621 (9)	
H32A	0.6542	−0.1420	0.3669	0.074*	
C33	0.8234 (3)	−0.1438 (3)	0.33179 (13)	0.0565 (8)	
H33A	0.8115	−0.0568	0.3141	0.068*	
C34	0.9324 (3)	−0.2443 (3)	0.33278 (14)	0.0579 (8)	
H34A	1.0086	−0.2394	0.3156	0.069*	
C35	1.0205 (2)	−0.2322 (2)	0.47654 (13)	0.0401 (6)	
H35A	1.1002	−0.2324	0.4625	0.048*	
C36	0.9896 (3)	−0.3367 (2)	0.50413 (13)	0.0476 (7)	
H36A	1.0441	−0.4224	0.5120	0.057*	
C37	0.8657 (3)	−0.2966 (3)	0.51724 (13)	0.0485 (7)	
H37A	0.8202	−0.3501	0.5357	0.058*	
C38	0.8179 (2)	−0.1667 (2)	0.49801 (12)	0.0387 (6)	
H38A	0.7344	−0.1149	0.5015	0.046*	
C39	0.9138 (2)	−0.1247 (2)	0.47311 (11)	0.0325 (5)	
C40	0.9114 (2)	−0.0062 (2)	0.43831 (11)	0.0318 (5)	
H40A	0.9776	−0.0047	0.4147	0.038*	
C41	0.4761 (2)	0.3987 (2)	0.38948 (12)	0.0374 (6)	
C42	0.3594 (3)	0.4262 (3)	0.36656 (14)	0.0502 (7)	
H42A	0.3384	0.3685	0.3511	0.060*	
C43	0.2726 (3)	0.5395 (3)	0.36633 (17)	0.0661 (10)	
H43A	0.1936	0.5575	0.3509	0.079*	
C44	0.3032 (4)	0.6243 (3)	0.38865 (16)	0.0700 (11)	
H44A	0.2447	0.7003	0.3881	0.084*	
C45	0.4188 (4)	0.5995 (3)	0.41191 (16)	0.0678 (10)	
H45A	0.4387	0.6583	0.4269	0.081*	
C46	0.5070 (3)	0.4849 (3)	0.41299 (15)	0.0560 (8)	
H46A	0.5855	0.4667	0.4293	0.067*	
C47	0.5434 (2)	0.1813 (2)	0.33622 (12)	0.0350 (5)	
C48	0.5855 (3)	0.1858 (3)	0.27625 (13)	0.0453 (7)	
H48A	0.6389	0.2277	0.2630	0.054*	
C49	0.5481 (3)	0.1280 (3)	0.23611 (14)	0.0558 (8)	
H49A	0.5774	0.1301	0.1962	0.067*	
C50	0.4684 (3)	0.0678 (3)	0.25496 (15)	0.0529 (8)	
H50A	0.4423	0.0305	0.2275	0.063*	
C51	0.4264 (3)	0.0622 (3)	0.31447 (16)	0.0586 (8)	
H51A	0.3726	0.0205	0.3271	0.070*	
C52	0.7240 (2)	0.2877 (2)	0.36106 (12)	0.0349 (5)	
C53	0.4636 (3)	0.1179 (3)	0.35530 (14)	0.0483 (7)	
H53A	0.4355	0.1132	0.3956	0.058*	
C54	0.7217 (3)	0.3907 (2)	0.31455 (13)	0.0464 (7)	
H54A	0.6484	0.4464	0.2948	0.056*	
C55	0.8287 (3)	0.4093 (3)	0.29803 (14)	0.0529 (8)	
H55A	0.8278	0.4774	0.2668	0.064*	
C56	0.9365 (3)	0.3276 (3)	0.32769 (14)	0.0484 (7)	
H56A	1.0079	0.3424	0.3169	0.058*	
C57	0.9415 (2)	0.2240 (2)	0.37321 (12)	0.0381 (6)	
H57A	1.0153	0.1686	0.3924	0.046*	
C58	0.8343 (2)	0.2040 (2)	0.38975 (11)	0.0317 (5)	
C59	0.6363 (4)	0.4203 (4)	0.07851 (19)	0.0871 (13)	
H59A	0.6183	0.3481	0.0747	0.105*	
H59B	0.6870	0.4376	0.0447	0.105*	
Cl5	0.71545 (16)	0.38869 (11)	0.15051 (6)	0.1188 (5)	
Cl6	0.50114 (12)	0.54512 (12)	0.06999 (6)	0.1046 (4)	

### Atomic displacement parameters (Å^2^)

**Table:**

	*U*^11^	*U*^22^	*U*^33^	*U*^12^	*U*^13^	*U*^23^
Pd1	0.03078 (10)	0.03280 (10)	0.02863 (9)	−0.01415 (8)	0.00142 (8)	−0.00840 (7)
Fe1	0.0412 (2)	0.03421 (19)	0.0382 (2)	−0.01154 (16)	0.00134 (17)	−0.01045 (16)
Cl1	0.0549 (4)	0.0684 (5)	0.0497 (4)	−0.0415 (4)	0.0038 (3)	−0.0178 (4)
Cl2	0.0504 (4)	0.0521 (4)	0.0337 (3)	−0.0280 (3)	0.0081 (3)	−0.0149 (3)
P1	0.0345 (3)	0.0375 (3)	0.0297 (3)	−0.0154 (3)	0.0023 (3)	−0.0068 (3)
N1	0.0300 (10)	0.0319 (10)	0.0313 (10)	−0.0115 (8)	0.0013 (9)	−0.0088 (9)
C1	0.091 (7)	0.057 (8)	0.042 (6)	−0.022 (6)	−0.008 (5)	−0.023 (6)
C2	0.072 (8)	0.057 (6)	0.040 (5)	−0.021 (5)	−0.001 (6)	−0.017 (4)
C3	0.077 (7)	0.063 (7)	0.038 (7)	−0.022 (6)	0.014 (5)	−0.008 (5)
C4	0.059 (7)	0.055 (6)	0.059 (6)	−0.003 (5)	0.029 (5)	−0.006 (4)
C5	0.075 (9)	0.038 (5)	0.056 (6)	−0.019 (6)	0.008 (7)	−0.021 (4)
C5'	0.077 (8)	0.096 (10)	0.060 (8)	−0.040 (7)	0.004 (6)	−0.046 (7)
C1'	0.114 (12)	0.048 (8)	0.055 (8)	0.006 (6)	−0.025 (7)	−0.026 (7)
C2'	0.140 (15)	0.067 (8)	0.032 (7)	−0.052 (10)	0.011 (10)	−0.010 (6)
C3'	0.067 (7)	0.111 (12)	0.065 (9)	−0.044 (9)	0.038 (6)	−0.044 (9)
C4'	0.053 (6)	0.049 (5)	0.062 (6)	−0.001 (5)	0.000 (6)	−0.028 (4)
C6	0.0442 (16)	0.0439 (15)	0.0409 (14)	−0.0097 (12)	−0.0097 (13)	−0.0083 (12)
C7	0.0568 (18)	0.0366 (14)	0.0397 (14)	−0.0149 (13)	0.0054 (13)	−0.0035 (12)
C8	0.0411 (15)	0.0388 (14)	0.0475 (15)	−0.0165 (12)	0.0100 (13)	−0.0090 (12)
C9	0.0347 (13)	0.0334 (12)	0.0372 (13)	−0.0131 (10)	0.0048 (11)	−0.0117 (11)
C10	0.0395 (14)	0.0357 (13)	0.0404 (14)	−0.0140 (11)	−0.0003 (12)	−0.0118 (11)
C11	0.0272 (12)	0.0367 (13)	0.0402 (13)	−0.0125 (10)	0.0018 (11)	−0.0146 (11)
C12	0.0475 (16)	0.0450 (15)	0.0323 (12)	−0.0246 (13)	0.0086 (12)	−0.0096 (11)
C13	0.0600 (19)	0.0469 (16)	0.0421 (15)	−0.0191 (14)	0.0053 (14)	−0.0125 (13)
C14	0.094 (3)	0.0428 (17)	0.0472 (17)	−0.0256 (17)	0.0077 (18)	−0.0127 (14)
C15	0.110 (3)	0.060 (2)	0.056 (2)	−0.050 (2)	0.026 (2)	−0.0174 (17)
C16	0.085 (3)	0.084 (3)	0.079 (3)	−0.058 (2)	0.034 (2)	−0.026 (2)
C17	0.0557 (19)	0.0593 (19)	0.0572 (18)	−0.0298 (16)	0.0132 (16)	−0.0142 (15)
C18	0.0392 (14)	0.0421 (14)	0.0347 (13)	−0.0151 (12)	0.0045 (11)	−0.0097 (11)
C19	0.0436 (16)	0.0610 (18)	0.0435 (15)	−0.0076 (14)	−0.0060 (13)	−0.0175 (14)
C20	0.0411 (17)	0.074 (2)	0.067 (2)	−0.0007 (16)	−0.0041 (16)	−0.0222 (18)
C21	0.0497 (19)	0.067 (2)	0.060 (2)	−0.0025 (16)	0.0122 (16)	−0.0233 (17)
C22	0.067 (2)	0.068 (2)	0.0394 (16)	−0.0139 (17)	0.0088 (15)	−0.0220 (15)
C23	0.0446 (16)	0.0554 (17)	0.0370 (14)	−0.0105 (13)	−0.0009 (13)	−0.0126 (13)
C24	0.0340 (13)	0.0352 (13)	0.0347 (12)	−0.0114 (10)	−0.0004 (11)	−0.0102 (11)
C25	0.0327 (13)	0.0329 (12)	0.0316 (12)	−0.0067 (10)	−0.0003 (10)	−0.0135 (10)
C26	0.0366 (14)	0.0407 (14)	0.0432 (14)	−0.0130 (11)	−0.0016 (12)	−0.0144 (12)
C27	0.0427 (16)	0.0497 (16)	0.0534 (17)	−0.0118 (13)	−0.0117 (14)	−0.0177 (14)
C28	0.0559 (19)	0.0476 (16)	0.0405 (15)	−0.0126 (14)	−0.0141 (14)	−0.0063 (13)
C29	0.0489 (17)	0.0445 (15)	0.0376 (14)	−0.0154 (13)	−0.0050 (13)	−0.0054 (12)
Pd2	0.02569 (9)	0.03334 (10)	0.02880 (9)	−0.00823 (7)	0.00318 (7)	−0.00707 (7)
Fe2	0.0342 (2)	0.03561 (19)	0.03562 (19)	−0.01031 (15)	−0.00046 (16)	−0.01028 (16)
Cl3	0.0351 (3)	0.0584 (4)	0.0316 (3)	−0.0102 (3)	0.0005 (3)	−0.0072 (3)
Cl4	0.0306 (3)	0.0624 (4)	0.0500 (4)	−0.0132 (3)	0.0117 (3)	−0.0112 (3)
P2	0.0291 (3)	0.0325 (3)	0.0317 (3)	−0.0071 (3)	0.0007 (3)	−0.0071 (3)
N2	0.0265 (10)	0.0340 (10)	0.0284 (10)	−0.0111 (8)	0.0023 (8)	−0.0072 (8)
C30	0.071 (2)	0.0550 (19)	0.0571 (19)	−0.0129 (17)	−0.0044 (17)	−0.0290 (16)
C31	0.082 (3)	0.088 (3)	0.0541 (19)	−0.051 (2)	−0.0042 (19)	−0.0248 (19)
C32	0.0404 (17)	0.096 (3)	0.0483 (17)	−0.0141 (17)	−0.0067 (15)	−0.0265 (18)
C33	0.067 (2)	0.0580 (19)	0.0365 (15)	−0.0111 (16)	−0.0058 (15)	−0.0102 (14)
C34	0.0531 (19)	0.076 (2)	0.0442 (16)	−0.0153 (17)	0.0106 (15)	−0.0239 (16)
C35	0.0334 (14)	0.0391 (14)	0.0453 (15)	−0.0075 (11)	−0.0049 (12)	−0.0118 (12)
C36	0.0584 (19)	0.0336 (14)	0.0430 (15)	−0.0083 (13)	−0.0117 (14)	−0.0040 (12)
C37	0.074 (2)	0.0449 (16)	0.0365 (14)	−0.0341 (15)	0.0017 (14)	−0.0068 (12)
C38	0.0431 (15)	0.0413 (14)	0.0361 (13)	−0.0181 (12)	0.0055 (12)	−0.0119 (11)
C39	0.0316 (13)	0.0315 (12)	0.0349 (12)	−0.0098 (10)	0.0003 (11)	−0.0097 (10)
C40	0.0252 (12)	0.0359 (12)	0.0352 (12)	−0.0108 (10)	0.0036 (10)	−0.0100 (10)
C41	0.0381 (14)	0.0339 (13)	0.0330 (12)	−0.0031 (11)	0.0011 (11)	−0.0078 (11)
C42	0.0399 (16)	0.0480 (16)	0.0550 (17)	−0.0031 (13)	0.0025 (14)	−0.0153 (14)
C43	0.0454 (18)	0.060 (2)	0.073 (2)	0.0055 (16)	0.0057 (17)	−0.0119 (18)
C44	0.079 (3)	0.0480 (19)	0.057 (2)	0.0105 (18)	0.0213 (19)	−0.0129 (16)
C45	0.099 (3)	0.0437 (18)	0.062 (2)	−0.0167 (19)	0.015 (2)	−0.0281 (16)
C46	0.060 (2)	0.0504 (18)	0.0571 (18)	−0.0113 (15)	−0.0046 (16)	−0.0216 (15)
C47	0.0331 (13)	0.0316 (12)	0.0363 (13)	−0.0049 (10)	−0.0006 (11)	−0.0090 (11)
C48	0.0430 (16)	0.0543 (17)	0.0416 (15)	−0.0185 (13)	0.0072 (13)	−0.0148 (13)
C49	0.062 (2)	0.064 (2)	0.0407 (15)	−0.0144 (16)	0.0064 (15)	−0.0211 (15)
C50	0.0586 (19)	0.0491 (17)	0.0511 (17)	−0.0116 (15)	−0.0094 (15)	−0.0218 (14)
C51	0.067 (2)	0.0591 (19)	0.063 (2)	−0.0366 (17)	0.0033 (17)	−0.0167 (16)
C52	0.0355 (13)	0.0341 (13)	0.0355 (13)	−0.0119 (11)	0.0040 (11)	−0.0085 (11)
C53	0.0536 (18)	0.0571 (18)	0.0431 (15)	−0.0284 (15)	0.0094 (14)	−0.0153 (14)
C54	0.0491 (17)	0.0380 (14)	0.0444 (15)	−0.0122 (13)	−0.0003 (13)	0.0013 (12)
C55	0.063 (2)	0.0476 (17)	0.0489 (17)	−0.0286 (15)	0.0071 (16)	0.0022 (14)
C56	0.0493 (17)	0.0520 (17)	0.0510 (17)	−0.0285 (14)	0.0126 (14)	−0.0095 (14)
C57	0.0345 (14)	0.0440 (14)	0.0391 (13)	−0.0159 (11)	0.0056 (11)	−0.0125 (12)
C58	0.0341 (13)	0.0314 (12)	0.0322 (12)	−0.0125 (10)	0.0056 (10)	−0.0106 (10)
C59	0.116 (4)	0.067 (2)	0.078 (3)	−0.021 (2)	0.005 (3)	−0.031 (2)
Cl5	0.1863 (15)	0.0860 (8)	0.0771 (7)	−0.0563 (9)	−0.0265 (8)	0.0120 (6)
Cl6	0.0972 (9)	0.0995 (8)	0.1150 (10)	−0.0202 (7)	0.0251 (7)	−0.0430 (7)

### Geometric parameters (Å, °)

**Table:**

Pd1—N1	2.051 (2)	C27—H27A	0.9300
Pd1—P1	2.2118 (6)	C28—C29	1.366 (4)
Pd1—Cl1	2.2832 (7)	C28—H28A	0.9300
Pd1—Cl2	2.3926 (6)	C29—H29A	0.9300
Fe1—C8	2.014 (3)	Pd2—N2	2.0616 (18)
Fe1—C1'	2.023 (8)	Pd2—P2	2.1979 (6)
Fe1—C5'	2.028 (7)	Pd2—Cl4	2.2875 (6)
Fe1—C9	2.028 (2)	Pd2—Cl3	2.3843 (7)
Fe1—C4	2.039 (10)	Fe2—C30	2.031 (3)
Fe1—C5	2.044 (8)	Fe2—C31	2.031 (3)
Fe1—C10	2.048 (2)	Fe2—C32	2.035 (3)
Fe1—C2'	2.049 (9)	Fe2—C38	2.040 (3)
Fe1—C4'	2.056 (8)	Fe2—C35	2.042 (3)
Fe1—C7	2.061 (3)	Fe2—C39	2.045 (2)
Fe1—C3	2.063 (9)	Fe2—C34	2.049 (3)
Fe1—C6	2.065 (3)	Fe2—C37	2.050 (3)
P1—C18	1.810 (3)	Fe2—C33	2.052 (3)
P1—C12	1.811 (3)	Fe2—C36	2.055 (3)
P1—C24	1.812 (3)	P2—C47	1.805 (3)
N1—C11	1.295 (3)	P2—C52	1.809 (3)
N1—C25	1.443 (3)	P2—C41	1.810 (2)
C1—C2	1.4200	N2—C40	1.294 (3)
C1—C5	1.4200	N2—C58	1.439 (3)
C1—H1A	0.9800	C30—C31	1.391 (5)
C2—C3	1.4200	C30—C34	1.391 (5)
C2—H2A	0.9800	C30—H30A	0.9800
C3—C4	1.4200	C31—C32	1.406 (5)
C3—H3A	0.9800	C31—H31A	0.9800
C4—C5	1.4200	C32—C33	1.386 (5)
C4—H4A	0.9800	C32—H32A	0.9800
C5—H5A	0.9800	C33—C34	1.424 (4)
C5'—C1'	1.4200	C33—H33A	0.9800
C5'—C4'	1.4200	C34—H34A	0.9800
C5'—H5'A	0.9800	C35—C36	1.413 (4)
C1'—C2'	1.4200	C35—C39	1.446 (3)
C1'—H1'A	0.9800	C35—H35A	0.9800
C2'—C3'	1.4200	C36—C37	1.418 (4)
C2'—H2'A	0.9800	C36—H36A	0.9800
C3'—C4'	1.4200	C37—C38	1.415 (4)
C3'—H3'A	0.9800	C37—H37A	0.9800
C4'—H4'A	0.9800	C38—C39	1.433 (3)
C6—C10	1.409 (4)	C38—H38A	0.9800
C6—C7	1.414 (4)	C39—C40	1.432 (3)
C6—H6A	0.9800	C40—H40A	0.9300
C7—C8	1.410 (4)	C41—C42	1.372 (4)
C7—H7A	0.9800	C41—C46	1.392 (4)
C8—C9	1.444 (3)	C42—C43	1.388 (4)
C8—H8A	0.9800	C42—H42A	0.9300
C9—C11	1.435 (4)	C43—C44	1.361 (5)
C9—C10	1.440 (4)	C43—H43A	0.9300
C10—H10A	0.9800	C44—C45	1.369 (5)
C11—H11A	0.9300	C44—H44A	0.9300
C12—C17	1.384 (4)	C45—C46	1.402 (4)
C12—C13	1.393 (4)	C45—H45A	0.9300
C13—C14	1.384 (4)	C46—H46A	0.9300
C13—H13A	0.9300	C47—C48	1.388 (4)
C14—C15	1.363 (5)	C47—C53	1.394 (4)
C14—H14A	0.9300	C48—C49	1.386 (4)
C15—C16	1.361 (5)	C48—H48A	0.9300
C15—H15A	0.9300	C49—C50	1.366 (4)
C16—C17	1.394 (4)	C49—H49A	0.9300
C16—H16A	0.9300	C50—C51	1.378 (4)
C17—H17A	0.9300	C50—H50A	0.9300
C18—C19	1.383 (4)	C51—C53	1.377 (4)
C18—C23	1.391 (4)	C51—H51A	0.9300
C19—C20	1.388 (4)	C52—C58	1.392 (3)
C19—H19A	0.9300	C52—C54	1.395 (3)
C20—C21	1.373 (5)	C53—H53A	0.9300
C20—H20A	0.9300	C54—C55	1.379 (4)
C21—C22	1.371 (5)	C54—H54A	0.9300
C21—H21A	0.9300	C55—C56	1.372 (4)
C22—C23	1.380 (4)	C55—H55A	0.9300
C22—H22A	0.9300	C56—C57	1.382 (4)
C23—H23A	0.9300	C56—H56A	0.9300
C24—C25	1.398 (3)	C57—C58	1.387 (3)
C24—C29	1.398 (4)	C57—H57A	0.9300
C25—C26	1.388 (4)	C59—Cl5	1.737 (4)
C26—C27	1.373 (4)	C59—Cl6	1.749 (4)
C26—H26A	0.9300	C59—H59A	0.9700
C27—C28	1.394 (4)	C59—H59B	0.9700
			
N1—Pd1—P1	81.09 (6)	C23—C22—H22A	119.8
N1—Pd1—Cl1	174.86 (6)	C22—C23—C18	119.9 (3)
P1—Pd1—Cl1	93.85 (3)	C22—C23—H23A	120.0
N1—Pd1—Cl2	92.77 (6)	C18—C23—H23A	120.0
P1—Pd1—Cl2	169.16 (3)	C25—C24—C29	119.1 (2)
Cl1—Pd1—Cl2	92.36 (2)	C25—C24—P1	113.86 (18)
C8—Fe1—C1'	115.4 (4)	C29—C24—P1	127.0 (2)
C8—Fe1—C5'	105.1 (2)	C26—C25—C24	120.7 (2)
C1'—Fe1—C5'	41.04 (13)	C26—C25—N1	123.8 (2)
C8—Fe1—C9	41.87 (9)	C24—C25—N1	115.4 (2)
C1'—Fe1—C9	108.4 (2)	C27—C26—C25	119.1 (3)
C5'—Fe1—C9	128.8 (3)	C27—C26—H26A	120.5
C8—Fe1—C4	150.5 (4)	C25—C26—H26A	120.5
C1'—Fe1—C4	71.4 (3)	C26—C27—C28	120.8 (3)
C5'—Fe1—C4	59.8 (3)	C26—C27—H27A	119.6
C9—Fe1—C4	167.0 (4)	C28—C27—H27A	119.6
C8—Fe1—C5	117.3 (3)	C29—C28—C27	120.2 (3)
C1'—Fe1—C5	55.2 (2)	C29—C28—H28A	119.9
C5'—Fe1—C5	22.0 (2)	C27—C28—H28A	119.9
C9—Fe1—C5	149.8 (4)	C28—C29—C24	120.1 (3)
C4—Fe1—C5	40.70 (16)	C28—C29—H29A	120.0
C8—Fe1—C10	69.71 (11)	C24—C29—H29A	120.0
C1'—Fe1—C10	131.9 (4)	N2—Pd2—P2	82.08 (6)
C5'—Fe1—C10	169.5 (4)	N2—Pd2—Cl4	171.66 (6)
C9—Fe1—C10	41.38 (10)	P2—Pd2—Cl4	90.52 (3)
C4—Fe1—C10	129.0 (4)	N2—Pd2—Cl3	93.96 (6)
C5—Fe1—C10	168.5 (4)	P2—Pd2—Cl3	166.02 (3)
C8—Fe1—C2'	150.4 (6)	Cl4—Pd2—Cl3	94.13 (2)
C1'—Fe1—C2'	40.81 (16)	C30—Fe2—C31	40.03 (14)
C5'—Fe1—C2'	68.62 (18)	C30—Fe2—C32	67.64 (14)
C9—Fe1—C2'	118.6 (4)	C31—Fe2—C32	40.45 (14)
C4—Fe1—C2'	52.5 (3)	C30—Fe2—C38	158.03 (13)
C5—Fe1—C2'	67.8 (3)	C31—Fe2—C38	121.46 (13)
C10—Fe1—C2'	111.1 (2)	C32—Fe2—C38	105.75 (12)
C8—Fe1—C4'	126.8 (4)	C30—Fe2—C35	123.09 (13)
C1'—Fe1—C4'	68.56 (15)	C31—Fe2—C35	156.33 (14)
C5'—Fe1—C4'	40.69 (12)	C32—Fe2—C35	162.72 (14)
C9—Fe1—C4'	166.9 (4)	C38—Fe2—C35	69.23 (11)
C4—Fe1—C4'	25.7 (3)	C30—Fe2—C39	159.77 (13)
C5—Fe1—C4'	18.8 (2)	C31—Fe2—C39	159.33 (14)
C10—Fe1—C4'	149.7 (4)	C32—Fe2—C39	124.13 (13)
C2'—Fe1—C4'	68.1 (2)	C38—Fe2—C39	41.07 (10)
C8—Fe1—C7	40.48 (11)	C35—Fe2—C39	41.44 (9)
C1'—Fe1—C7	147.9 (5)	C30—Fe2—C34	39.86 (13)
C5'—Fe1—C7	114.4 (3)	C31—Fe2—C34	67.33 (14)
C9—Fe1—C7	68.58 (10)	C32—Fe2—C34	67.72 (13)
C4—Fe1—C7	118.8 (3)	C38—Fe2—C34	159.09 (12)
C5—Fe1—C7	110.7 (2)	C35—Fe2—C34	110.78 (13)
C10—Fe1—C7	67.99 (11)	C39—Fe2—C34	124.91 (12)
C2'—Fe1—C7	168.9 (6)	C30—Fe2—C37	122.86 (13)
C4'—Fe1—C7	106.8 (2)	C31—Fe2—C37	105.24 (13)
C8—Fe1—C3	167.6 (4)	C32—Fe2—C37	119.37 (14)
C1'—Fe1—C3	57.1 (4)	C38—Fe2—C37	40.49 (10)
C5'—Fe1—C3	75.9 (3)	C35—Fe2—C37	68.22 (12)
C9—Fe1—C3	128.0 (3)	C39—Fe2—C37	68.36 (10)
C4—Fe1—C3	40.49 (17)	C34—Fe2—C37	160.23 (13)
C5—Fe1—C3	68.0 (2)	C30—Fe2—C33	67.32 (13)
C10—Fe1—C3	107.1 (2)	C31—Fe2—C33	67.12 (14)
C2'—Fe1—C3	18.3 (4)	C32—Fe2—C33	39.65 (13)
C4'—Fe1—C3	61.9 (3)	C38—Fe2—C33	121.83 (12)
C7—Fe1—C3	150.6 (3)	C35—Fe2—C33	127.98 (13)
C8—Fe1—C6	68.43 (12)	C39—Fe2—C33	110.05 (12)
C1'—Fe1—C6	170.8 (5)	C34—Fe2—C33	40.63 (12)
C5'—Fe1—C6	147.7 (4)	C37—Fe2—C33	155.35 (13)
C9—Fe1—C6	68.36 (11)	C30—Fe2—C36	108.03 (13)
C4—Fe1—C6	109.7 (3)	C31—Fe2—C36	120.10 (14)
C5—Fe1—C6	131.7 (4)	C32—Fe2—C36	154.77 (14)
C10—Fe1—C6	40.07 (10)	C38—Fe2—C36	68.50 (11)
C2'—Fe1—C6	132.3 (4)	C35—Fe2—C36	40.33 (11)
C4'—Fe1—C6	116.6 (2)	C39—Fe2—C36	68.59 (10)
C7—Fe1—C6	40.09 (11)	C34—Fe2—C36	125.94 (13)
C3—Fe1—C6	117.4 (2)	C37—Fe2—C36	40.43 (12)
C18—P1—C12	104.83 (12)	C33—Fe2—C36	163.81 (13)
C18—P1—C24	107.78 (12)	C47—P2—C52	107.99 (12)
C12—P1—C24	108.48 (12)	C47—P2—C41	108.37 (12)
C18—P1—Pd1	116.41 (9)	C52—P2—C41	105.87 (12)
C12—P1—Pd1	119.66 (9)	C47—P2—Pd2	117.30 (8)
C24—P1—Pd1	98.89 (8)	C52—P2—Pd2	98.89 (8)
C11—N1—C25	119.1 (2)	C41—P2—Pd2	117.02 (8)
C11—N1—Pd1	126.51 (17)	C40—N2—C58	118.00 (19)
C25—N1—Pd1	114.20 (15)	C40—N2—Pd2	128.02 (16)
C2—C1—C5	108.0	C58—N2—Pd2	113.58 (14)
C2—C1—Fe1	70.5 (3)	C31—C30—C34	108.8 (3)
C5—C1—Fe1	68.8 (3)	C31—C30—Fe2	69.99 (19)
C2—C1—H1A	126.0	C34—C30—Fe2	70.77 (18)
C5—C1—H1A	126.0	C31—C30—H30A	125.6
Fe1—C1—H1A	126.0	C34—C30—H30A	125.6
C3—C2—C1	108.0	Fe2—C30—H30A	125.6
C3—C2—Fe1	69.2 (4)	C30—C31—C32	108.1 (3)
C1—C2—Fe1	69.6 (3)	C30—C31—Fe2	69.97 (19)
C3—C2—H2A	126.0	C32—C31—Fe2	69.90 (19)
C1—C2—H2A	126.0	C30—C31—H31A	126.0
Fe1—C2—H2A	126.0	C32—C31—H31A	126.0
C4—C3—C2	108.0	Fe2—C31—H31A	126.0
C4—C3—Fe1	68.8 (3)	C33—C32—C31	107.9 (3)
C2—C3—Fe1	70.7 (3)	C33—C32—Fe2	70.86 (19)
C4—C3—H3A	126.0	C31—C32—Fe2	69.65 (19)
C2—C3—H3A	126.0	C33—C32—H32A	126.0
Fe1—C3—H3A	126.0	C31—C32—H32A	126.0
C3—C4—C5	108.0	Fe2—C32—H32A	126.0
C3—C4—Fe1	70.7 (3)	C32—C33—C34	108.1 (3)
C5—C4—Fe1	69.8 (3)	C32—C33—Fe2	69.49 (18)
C3—C4—H4A	126.0	C34—C33—Fe2	69.57 (17)
C5—C4—H4A	126.0	C32—C33—H33A	125.9
Fe1—C4—H4A	126.0	C34—C33—H33A	125.9
C4—C5—C1	108.0	Fe2—C33—H33A	125.9
C4—C5—Fe1	69.5 (4)	C30—C34—C33	107.0 (3)
C1—C5—Fe1	70.8 (3)	C30—C34—Fe2	69.37 (18)
C4—C5—H5A	126.0	C33—C34—Fe2	69.81 (16)
C1—C5—H5A	126.0	C30—C34—H34A	126.5
Fe1—C5—H5A	126.0	C33—C34—H34A	126.5
C1'—C5'—C4'	108.0	Fe2—C34—H34A	126.5
C1'—C5'—Fe1	69.3 (3)	C36—C35—C39	107.8 (2)
C4'—C5'—Fe1	70.7 (4)	C36—C35—Fe2	70.34 (16)
C1'—C5'—H5'A	126.0	C39—C35—Fe2	69.39 (14)
C4'—C5'—H5'A	126.0	C36—C35—H35A	126.1
Fe1—C5'—H5'A	126.0	C39—C35—H35A	126.1
C5'—C1'—C2'	108.0	Fe2—C35—H35A	126.1
C5'—C1'—Fe1	69.6 (3)	C35—C36—C37	108.3 (2)
C2'—C1'—Fe1	70.6 (4)	C35—C36—Fe2	69.33 (15)
C5'—C1'—H1'A	126.0	C37—C36—Fe2	69.57 (16)
C2'—C1'—H1'A	126.0	C35—C36—H36A	125.9
Fe1—C1'—H1'A	126.0	C37—C36—H36A	125.9
C1'—C2'—C3'	108.0	Fe2—C36—H36A	125.9
C1'—C2'—Fe1	68.6 (3)	C38—C37—C36	108.9 (2)
C3'—C2'—Fe1	70.6 (3)	C38—C37—Fe2	69.41 (15)
C1'—C2'—H2'A	126.0	C36—C37—Fe2	70.00 (16)
C3'—C2'—H2'A	126.0	C38—C37—H37A	125.6
Fe1—C2'—H2'A	126.0	C36—C37—H37A	125.6
C4'—C3'—C2'	108.0	Fe2—C37—H37A	125.6
C4'—C3'—Fe1	69.4 (3)	C37—C38—C39	107.7 (2)
C2'—C3'—Fe1	69.1 (3)	C37—C38—Fe2	70.10 (15)
C4'—C3'—H3'A	126.0	C39—C38—Fe2	69.63 (14)
C2'—C3'—H3'A	126.0	C37—C38—H38A	126.1
Fe1—C3'—H3'A	126.0	C39—C38—H38A	126.1
C3'—C4'—C5'	108.0	Fe2—C38—H38A	126.1
C3'—C4'—Fe1	70.3 (4)	C40—C39—C38	130.9 (2)
C5'—C4'—Fe1	68.6 (3)	C40—C39—C35	120.7 (2)
C3'—C4'—H4'A	126.0	C38—C39—C35	107.3 (2)
C5'—C4'—H4'A	126.0	C40—C39—Fe2	117.09 (17)
Fe1—C4'—H4'A	126.0	C38—C39—Fe2	69.30 (14)
C10—C6—C7	108.9 (2)	C35—C39—Fe2	69.17 (14)
C10—C6—Fe1	69.30 (15)	N2—C40—C39	127.6 (2)
C7—C6—Fe1	69.79 (16)	N2—C40—H40A	116.2
C10—C6—H6A	125.5	C39—C40—H40A	116.2
C7—C6—H6A	125.5	C42—C41—C46	119.6 (3)
Fe1—C6—H6A	125.5	C42—C41—P2	122.0 (2)
C8—C7—C6	108.6 (2)	C46—C41—P2	118.3 (2)
C8—C7—Fe1	67.96 (15)	C41—C42—C43	120.4 (3)
C6—C7—Fe1	70.12 (15)	C41—C42—H42A	119.8
C8—C7—H7A	125.7	C43—C42—H42A	119.8
C6—C7—H7A	125.7	C44—C43—C42	119.9 (3)
Fe1—C7—H7A	125.7	C44—C43—H43A	120.0
C7—C8—C9	107.6 (2)	C42—C43—H43A	120.0
C7—C8—Fe1	71.56 (16)	C43—C44—C45	121.0 (3)
C9—C8—Fe1	69.59 (14)	C43—C44—H44A	119.5
C7—C8—H8A	126.2	C45—C44—H44A	119.5
C9—C8—H8A	126.2	C44—C45—C46	119.6 (3)
Fe1—C8—H8A	126.2	C44—C45—H45A	120.2
C11—C9—C10	128.6 (2)	C46—C45—H45A	120.2
C11—C9—C8	121.9 (2)	C41—C46—C45	119.4 (3)
C10—C9—C8	107.2 (2)	C41—C46—H46A	120.3
C11—C9—Fe1	112.88 (17)	C45—C46—H46A	120.3
C10—C9—Fe1	70.06 (14)	C48—C47—C53	119.2 (3)
C8—C9—Fe1	68.54 (14)	C48—C47—P2	121.6 (2)
C6—C10—C9	107.6 (2)	C53—C47—P2	119.2 (2)
C6—C10—Fe1	70.63 (15)	C49—C48—C47	120.0 (3)
C9—C10—Fe1	68.57 (14)	C49—C48—H48A	120.0
C6—C10—H10A	126.2	C47—C48—H48A	120.0
C9—C10—H10A	126.2	C50—C49—C48	120.2 (3)
Fe1—C10—H10A	126.2	C50—C49—H49A	119.9
N1—C11—C9	126.1 (2)	C48—C49—H49A	119.9
N1—C11—H11A	116.9	C49—C50—C51	120.2 (3)
C9—C11—H11A	116.9	C49—C50—H50A	119.9
C17—C12—C13	119.4 (3)	C51—C50—H50A	119.9
C17—C12—P1	119.4 (2)	C53—C51—C50	120.4 (3)
C13—C12—P1	121.1 (2)	C53—C51—H51A	119.8
C14—C13—C12	120.0 (3)	C50—C51—H51A	119.8
C14—C13—H13A	120.0	C58—C52—C54	119.8 (2)
C12—C13—H13A	120.0	C58—C52—P2	114.71 (18)
C15—C14—C13	120.0 (3)	C54—C52—P2	125.5 (2)
C15—C14—H14A	120.0	C51—C53—C47	119.9 (3)
C13—C14—H14A	120.0	C51—C53—H53A	120.0
C16—C15—C14	120.6 (3)	C47—C53—H53A	120.0
C16—C15—H15A	119.7	C55—C54—C52	119.6 (3)
C14—C15—H15A	119.7	C55—C54—H54A	120.2
C15—C16—C17	120.7 (4)	C52—C54—H54A	120.2
C15—C16—H16A	119.7	C56—C55—C54	120.0 (3)
C17—C16—H16A	119.7	C56—C55—H55A	120.0
C12—C17—C16	119.2 (3)	C54—C55—H55A	120.0
C12—C17—H17A	120.4	C55—C56—C57	121.5 (3)
C16—C17—H17A	120.4	C55—C56—H56A	119.3
C19—C18—C23	119.2 (3)	C57—C56—H56A	119.3
C19—C18—P1	119.6 (2)	C56—C57—C58	118.8 (3)
C23—C18—P1	121.0 (2)	C56—C57—H57A	120.6
C18—C19—C20	120.2 (3)	C58—C57—H57A	120.6
C18—C19—H19A	119.9	C57—C58—C52	120.3 (2)
C20—C19—H19A	119.9	C57—C58—N2	123.9 (2)
C21—C20—C19	120.0 (3)	C52—C58—N2	115.8 (2)
C21—C20—H20A	120.0	Cl5—C59—Cl6	112.5 (2)
C19—C20—H20A	120.0	Cl5—C59—H59A	109.1
C22—C21—C20	120.1 (3)	Cl6—C59—H59A	109.1
C22—C21—H21A	119.9	Cl5—C59—H59B	109.1
C20—C21—H21A	119.9	Cl6—C59—H59B	109.1
C21—C22—C23	120.5 (3)	H59A—C59—H59B	107.8
C21—C22—H22A	119.7		
			
N1—Pd1—P1—C18	84.82 (11)	Fe1—C6—C10—C9	−58.80 (17)
Cl1—Pd1—P1—C18	−94.30 (10)	C7—C6—C10—Fe1	58.7 (2)
Cl2—Pd1—P1—C18	140.89 (15)	C11—C9—C10—C6	164.0 (2)
N1—Pd1—P1—C12	−147.46 (12)	C8—C9—C10—C6	1.4 (3)
Cl1—Pd1—P1—C12	33.41 (11)	Fe1—C9—C10—C6	60.10 (18)
Cl2—Pd1—P1—C12	−91.39 (17)	C11—C9—C10—Fe1	103.9 (3)
N1—Pd1—P1—C24	−30.19 (10)	C8—C9—C10—Fe1	−58.69 (17)
Cl1—Pd1—P1—C24	150.68 (8)	C8—Fe1—C10—C6	−80.25 (17)
Cl2—Pd1—P1—C24	25.88 (16)	C1'—Fe1—C10—C6	173.3 (6)
P1—Pd1—N1—C11	−137.0 (2)	C5'—Fe1—C10—C6	−142.3 (15)
Cl2—Pd1—N1—C11	51.96 (19)	C9—Fe1—C10—C6	−118.9 (2)
P1—Pd1—N1—C25	38.35 (14)	C4—Fe1—C10—C6	73.2 (4)
Cl2—Pd1—N1—C25	−132.66 (15)	C5—Fe1—C10—C6	49.1 (10)
C8—Fe1—C1—C2	−130.1 (4)	C2'—Fe1—C10—C6	131.4 (6)
C1'—Fe1—C1—C2	−15.7 (12)	C4'—Fe1—C10—C6	49.1 (6)
C5'—Fe1—C1—C2	145.2 (8)	C7—Fe1—C10—C6	−36.74 (17)
C9—Fe1—C1—C2	−85.4 (4)	C3—Fe1—C10—C6	112.4 (4)
C4—Fe1—C1—C2	81.2 (2)	C8—Fe1—C10—C9	38.62 (15)
C5—Fe1—C1—C2	119.3 (2)	C1'—Fe1—C10—C9	−67.8 (6)
C10—Fe1—C1—C2	−48.8 (6)	C5'—Fe1—C10—C9	−23.4 (15)
C2'—Fe1—C1—C2	22.3 (5)	C4—Fe1—C10—C9	−168.0 (4)
C4'—Fe1—C1—C2	107.5 (4)	C5—Fe1—C10—C9	167.9 (9)
C7—Fe1—C1—C2	−169.3 (3)	C2'—Fe1—C10—C9	−109.7 (6)
C3—Fe1—C1—C2	37.27 (14)	C4'—Fe1—C10—C9	167.9 (5)
C8—Fe1—C1—C5	110.6 (4)	C7—Fe1—C10—C9	82.12 (16)
C1'—Fe1—C1—C5	−135.0 (12)	C3—Fe1—C10—C9	−128.7 (4)
C5'—Fe1—C1—C5	25.9 (8)	C6—Fe1—C10—C9	118.9 (2)
C9—Fe1—C1—C5	155.3 (4)	C25—N1—C11—C9	−164.5 (2)
C4—Fe1—C1—C5	−38.12 (15)	Pd1—N1—C11—C9	10.7 (3)
C10—Fe1—C1—C5	−168.1 (5)	C10—C9—C11—N1	26.4 (4)
C2'—Fe1—C1—C5	−97.0 (5)	C8—C9—C11—N1	−173.3 (2)
C4'—Fe1—C1—C5	−11.8 (3)	Fe1—C9—C11—N1	108.6 (2)
C7—Fe1—C1—C5	71.4 (4)	C18—P1—C12—C17	33.9 (3)
C3—Fe1—C1—C5	−82.0 (3)	C24—P1—C12—C17	148.8 (2)
C5—C1—C2—C3	0.0	Pd1—P1—C12—C17	−99.0 (2)
Fe1—C1—C2—C3	−58.7 (4)	C18—P1—C12—C13	−149.4 (2)
C5—C1—C2—Fe1	58.7 (4)	C24—P1—C12—C13	−34.5 (3)
C8—Fe1—C2—C3	−170.4 (4)	Pd1—P1—C12—C13	77.7 (2)
C1'—Fe1—C2—C3	133.1 (9)	C17—C12—C13—C14	0.4 (4)
C5'—Fe1—C2—C3	104.9 (4)	P1—C12—C13—C14	−176.3 (2)
C9—Fe1—C2—C3	−129.6 (5)	C12—C13—C14—C15	0.8 (5)
C4—Fe1—C2—C3	37.81 (16)	C13—C14—C15—C16	−1.3 (5)
C5—Fe1—C2—C3	82.0 (3)	C14—C15—C16—C17	0.6 (6)
C10—Fe1—C2—C3	−86.0 (5)	C13—C12—C17—C16	−1.1 (5)
C2'—Fe1—C2—C3	−4.0 (9)	P1—C12—C17—C16	175.6 (3)
C4'—Fe1—C2—C3	63.8 (3)	C15—C16—C17—C12	0.6 (5)
C7—Fe1—C2—C3	165.7 (9)	C12—P1—C18—C19	−91.5 (2)
C6—Fe1—C2—C3	−53.6 (6)	C24—P1—C18—C19	153.1 (2)
C8—Fe1—C2—C1	70.0 (4)	Pd1—P1—C18—C19	43.2 (3)
C1'—Fe1—C2—C1	13.5 (8)	C12—P1—C18—C23	84.9 (2)
C5'—Fe1—C2—C1	−14.7 (3)	C24—P1—C18—C23	−30.5 (3)
C9—Fe1—C2—C1	110.8 (5)	Pd1—P1—C18—C23	−140.4 (2)
C4—Fe1—C2—C1	−81.8 (2)	C23—C18—C19—C20	−1.1 (5)
C5—Fe1—C2—C1	−37.64 (15)	P1—C18—C19—C20	175.4 (3)
C10—Fe1—C2—C1	154.4 (4)	C18—C19—C20—C21	1.6 (5)
C2'—Fe1—C2—C1	−123.6 (9)	C19—C20—C21—C22	−0.7 (6)
C4'—Fe1—C2—C1	−55.8 (3)	C20—C21—C22—C23	−0.7 (6)
C7—Fe1—C2—C1	46.1 (10)	C21—C22—C23—C18	1.2 (5)
C3—Fe1—C2—C1	−119.60 (19)	C19—C18—C23—C22	−0.3 (4)
C6—Fe1—C2—C1	−173.2 (5)	P1—C18—C23—C22	−176.7 (2)
C1—C2—C3—C4	0.0	C18—P1—C24—C25	−97.19 (19)
Fe1—C2—C3—C4	−58.9 (3)	C12—P1—C24—C25	149.82 (18)
C1—C2—C3—Fe1	58.9 (3)	Pd1—P1—C24—C25	24.35 (18)
C8—Fe1—C3—C4	156.0 (12)	C18—P1—C24—C29	81.0 (3)
C1'—Fe1—C3—C4	100.0 (3)	C12—P1—C24—C29	−32.0 (3)
C5'—Fe1—C3—C4	59.7 (3)	Pd1—P1—C24—C29	−157.4 (2)
C9—Fe1—C3—C4	−171.6 (5)	C29—C24—C25—C26	1.6 (4)
C5—Fe1—C3—C4	38.15 (16)	P1—C24—C25—C26	180.00 (19)
C10—Fe1—C3—C4	−130.7 (5)	C29—C24—C25—N1	−176.4 (2)
C2'—Fe1—C3—C4	123.8 (11)	P1—C24—C25—N1	2.0 (3)
C4'—Fe1—C3—C4	18.5 (3)	C11—N1—C25—C26	−34.4 (3)
C7—Fe1—C3—C4	−55.3 (5)	Pd1—N1—C25—C26	149.9 (2)
C6—Fe1—C3—C4	−88.7 (4)	C11—N1—C25—C24	143.5 (2)
C8—Fe1—C3—C2	36.9 (12)	Pd1—N1—C25—C24	−32.2 (2)
C1'—Fe1—C3—C2	−19.2 (3)	C24—C25—C26—C27	−0.3 (4)
C5'—Fe1—C3—C2	−59.4 (3)	N1—C25—C26—C27	177.5 (2)
C9—Fe1—C3—C2	69.2 (5)	C25—C26—C27—C28	−1.1 (4)
C4—Fe1—C3—C2	−119.12 (17)	C26—C27—C28—C29	1.2 (4)
C5—Fe1—C3—C2	−81.0 (2)	C27—C28—C29—C24	0.2 (4)
C10—Fe1—C3—C2	110.2 (5)	C25—C24—C29—C28	−1.6 (4)
C2'—Fe1—C3—C2	4.7 (10)	P1—C24—C29—C28	−179.7 (2)
C4'—Fe1—C3—C2	−100.6 (3)	N2—Pd2—P2—C47	87.24 (11)
C7—Fe1—C3—C2	−174.4 (4)	Cl4—Pd2—P2—C47	−88.89 (10)
C6—Fe1—C3—C2	152.2 (4)	Cl3—Pd2—P2—C47	161.56 (12)
C2—C3—C4—C5	0.0	N2—Pd2—P2—C52	−28.38 (10)
Fe1—C3—C4—C5	−60.1 (3)	Cl4—Pd2—P2—C52	155.48 (8)
C2—C3—C4—Fe1	60.1 (3)	Cl3—Pd2—P2—C52	45.93 (13)
C8—Fe1—C4—C3	−169.8 (5)	N2—Pd2—P2—C41	−141.37 (12)
C1'—Fe1—C4—C3	−60.8 (3)	Cl4—Pd2—P2—C41	42.50 (11)
C5'—Fe1—C4—C3	−104.3 (3)	Cl3—Pd2—P2—C41	−67.06 (15)
C9—Fe1—C4—C3	30.7 (13)	P2—Pd2—N2—C40	−135.9 (2)
C5—Fe1—C4—C3	−118.54 (18)	Cl3—Pd2—N2—C40	57.5 (2)
C10—Fe1—C4—C3	68.7 (4)	P2—Pd2—N2—C58	36.60 (15)
C2'—Fe1—C4—C3	−19.2 (4)	Cl3—Pd2—N2—C58	−129.92 (15)
C4'—Fe1—C4—C3	−139.8 (7)	C32—Fe2—C30—C31	−37.8 (2)
C7—Fe1—C4—C3	152.6 (4)	C38—Fe2—C30—C31	38.6 (4)
C6—Fe1—C4—C3	109.5 (4)	C35—Fe2—C30—C31	157.5 (2)
C8—Fe1—C4—C5	−51.2 (5)	C39—Fe2—C30—C31	−167.5 (3)
C1'—Fe1—C4—C5	57.8 (4)	C34—Fe2—C30—C31	−119.4 (3)
C5'—Fe1—C4—C5	14.2 (3)	C37—Fe2—C30—C31	73.6 (3)
C9—Fe1—C4—C5	149.3 (12)	C33—Fe2—C30—C31	−80.9 (2)
C10—Fe1—C4—C5	−172.8 (4)	C36—Fe2—C30—C31	115.6 (2)
C2'—Fe1—C4—C5	99.3 (5)	C31—Fe2—C30—C34	119.4 (3)
C4'—Fe1—C4—C5	−21.2 (7)	C32—Fe2—C30—C34	81.5 (2)
C7—Fe1—C4—C5	−88.9 (4)	C38—Fe2—C30—C34	157.9 (3)
C3—Fe1—C4—C5	118.54 (18)	C35—Fe2—C30—C34	−83.1 (2)
C6—Fe1—C4—C5	−131.9 (4)	C39—Fe2—C30—C34	−48.1 (4)
C3—C4—C5—C1	0.0	C37—Fe2—C30—C34	−167.0 (2)
Fe1—C4—C5—C1	−60.6 (3)	C33—Fe2—C30—C34	38.5 (2)
C3—C4—C5—Fe1	60.6 (3)	C36—Fe2—C30—C34	−125.0 (2)
C2—C1—C5—C4	0.0	C34—C30—C31—C32	−0.6 (4)
Fe1—C1—C5—C4	59.8 (3)	Fe2—C30—C31—C32	59.8 (2)
C2—C1—C5—Fe1	−59.8 (3)	C34—C30—C31—Fe2	−60.4 (2)
C8—Fe1—C5—C4	154.4 (4)	C32—Fe2—C31—C30	119.0 (3)
C1'—Fe1—C5—C4	−102.4 (5)	C38—Fe2—C31—C30	−164.14 (19)
C5'—Fe1—C5—C4	−145.3 (8)	C35—Fe2—C31—C30	−53.0 (4)
C9—Fe1—C5—C4	−166.8 (5)	C39—Fe2—C31—C30	167.8 (3)
C10—Fe1—C5—C4	29.1 (11)	C34—Fe2—C31—C30	37.2 (2)
C2'—Fe1—C5—C4	−57.8 (4)	C37—Fe2—C31—C30	−123.4 (2)
C4'—Fe1—C5—C4	29.2 (9)	C33—Fe2—C31—C30	81.5 (2)
C7—Fe1—C5—C4	110.4 (4)	C36—Fe2—C31—C30	−82.3 (2)
C3—Fe1—C5—C4	−37.96 (16)	C30—Fe2—C31—C32	−119.0 (3)
C6—Fe1—C5—C4	69.8 (4)	C38—Fe2—C31—C32	76.9 (2)
C8—Fe1—C5—C1	−87.0 (4)	C35—Fe2—C31—C32	−172.0 (3)
C1'—Fe1—C5—C1	16.3 (5)	C39—Fe2—C31—C32	48.8 (4)
C5'—Fe1—C5—C1	−26.7 (8)	C34—Fe2—C31—C32	−81.8 (2)
C9—Fe1—C5—C1	−48.2 (5)	C37—Fe2—C31—C32	117.6 (2)
C4—Fe1—C5—C1	118.65 (16)	C33—Fe2—C31—C32	−37.52 (19)
C10—Fe1—C5—C1	147.8 (10)	C36—Fe2—C31—C32	158.70 (19)
C2'—Fe1—C5—C1	60.9 (5)	C30—C31—C32—C33	1.0 (4)
C4'—Fe1—C5—C1	147.8 (10)	Fe2—C31—C32—C33	60.8 (2)
C7—Fe1—C5—C1	−130.9 (4)	C30—C31—C32—Fe2	−59.8 (2)
C3—Fe1—C5—C1	80.7 (2)	C30—Fe2—C32—C33	−81.0 (2)
C6—Fe1—C5—C1	−171.6 (3)	C31—Fe2—C32—C33	−118.4 (3)
C8—Fe1—C5'—C1'	−111.5 (6)	C38—Fe2—C32—C33	121.24 (19)
C9—Fe1—C5'—C1'	−72.1 (5)	C35—Fe2—C32—C33	50.8 (5)
C4—Fe1—C5'—C1'	96.1 (4)	C39—Fe2—C32—C33	80.3 (2)
C5—Fe1—C5'—C1'	121.5 (7)	C34—Fe2—C32—C33	−37.7 (2)
C10—Fe1—C5'—C1'	−52.4 (11)	C37—Fe2—C32—C33	162.82 (18)
C2'—Fe1—C5'—C1'	37.99 (15)	C36—Fe2—C32—C33	−165.9 (3)
C4'—Fe1—C5'—C1'	118.8 (2)	C30—Fe2—C32—C31	37.5 (2)
C7—Fe1—C5'—C1'	−153.6 (6)	C38—Fe2—C32—C31	−120.3 (2)
C3—Fe1—C5'—C1'	55.7 (4)	C35—Fe2—C32—C31	169.2 (4)
C6—Fe1—C5'—C1'	175.1 (6)	C39—Fe2—C32—C31	−161.29 (19)
C8—Fe1—C5'—C4'	129.7 (6)	C34—Fe2—C32—C31	80.7 (2)
C1'—Fe1—C5'—C4'	−118.8 (2)	C37—Fe2—C32—C31	−78.7 (2)
C9—Fe1—C5'—C4'	169.0 (5)	C33—Fe2—C32—C31	118.4 (3)
C4—Fe1—C5'—C4'	−22.7 (4)	C36—Fe2—C32—C31	−47.5 (4)
C5—Fe1—C5'—C4'	2.7 (7)	C31—C32—C33—C34	−1.0 (4)
C10—Fe1—C5'—C4'	−171.3 (11)	Fe2—C32—C33—C34	59.0 (2)
C2'—Fe1—C5'—C4'	−80.8 (3)	C31—C32—C33—Fe2	−60.1 (2)
C7—Fe1—C5'—C4'	87.6 (5)	C30—Fe2—C33—C32	81.8 (2)
C3—Fe1—C5'—C4'	−63.1 (4)	C31—Fe2—C33—C32	38.3 (2)
C6—Fe1—C5'—C4'	56.3 (6)	C38—Fe2—C33—C32	−75.6 (2)
C4'—C5'—C1'—C2'	0.0	C35—Fe2—C33—C32	−163.04 (19)
Fe1—C5'—C1'—C2'	−60.4 (4)	C39—Fe2—C33—C32	−119.7 (2)
C4'—C5'—C1'—Fe1	60.4 (4)	C34—Fe2—C33—C32	119.6 (3)
C8—Fe1—C1'—C5'	83.9 (5)	C37—Fe2—C33—C32	−38.1 (4)
C9—Fe1—C1'—C5'	128.6 (5)	C36—Fe2—C33—C32	158.2 (4)
C4—Fe1—C1'—C5'	−65.1 (3)	C30—Fe2—C33—C34	−37.8 (2)
C5—Fe1—C1'—C5'	−22.9 (3)	C31—Fe2—C33—C34	−81.3 (2)
C10—Fe1—C1'—C5'	168.8 (4)	C32—Fe2—C33—C34	−119.6 (3)
C2'—Fe1—C1'—C5'	−118.7 (2)	C38—Fe2—C33—C34	164.79 (19)
C4'—Fe1—C1'—C5'	−37.86 (15)	C35—Fe2—C33—C34	77.4 (2)
C7—Fe1—C1'—C5'	49.8 (6)	C39—Fe2—C33—C34	120.7 (2)
C3—Fe1—C1'—C5'	−107.5 (4)	C37—Fe2—C33—C34	−157.7 (3)
C8—Fe1—C1'—C2'	−157.4 (6)	C36—Fe2—C33—C34	38.6 (5)
C5'—Fe1—C1'—C2'	118.7 (2)	C31—C30—C34—C33	0.0 (4)
C9—Fe1—C1'—C2'	−112.7 (6)	Fe2—C30—C34—C33	−59.9 (2)
C4—Fe1—C1'—C2'	53.7 (4)	C31—C30—C34—Fe2	59.9 (2)
C5—Fe1—C1'—C2'	95.9 (3)	C32—C33—C34—C30	0.7 (4)
C10—Fe1—C1'—C2'	−72.5 (4)	Fe2—C33—C34—C30	59.6 (2)
C4'—Fe1—C1'—C2'	80.9 (3)	C32—C33—C34—Fe2	−59.0 (2)
C7—Fe1—C1'—C2'	168.5 (7)	C31—Fe2—C34—C30	−37.4 (2)
C3—Fe1—C1'—C2'	11.2 (5)	C32—Fe2—C34—C30	−81.3 (2)
C5'—C1'—C2'—C3'	0.0	C38—Fe2—C34—C30	−156.8 (3)
Fe1—C1'—C2'—C3'	−59.8 (3)	C35—Fe2—C34—C30	117.2 (2)
C5'—C1'—C2'—Fe1	59.8 (3)	C39—Fe2—C34—C30	161.70 (19)
C8—Fe1—C2'—C1'	44.7 (7)	C37—Fe2—C34—C30	33.9 (5)
C5'—Fe1—C2'—C1'	−38.20 (17)	C33—Fe2—C34—C30	−118.2 (3)
C9—Fe1—C2'—C1'	85.4 (5)	C36—Fe2—C34—C30	74.2 (2)
C4—Fe1—C2'—C1'	−105.9 (4)	C30—Fe2—C34—C33	118.2 (3)
C5—Fe1—C2'—C1'	−61.9 (3)	C31—Fe2—C34—C33	80.8 (2)
C10—Fe1—C2'—C1'	130.5 (5)	C32—Fe2—C34—C33	36.8 (2)
C4'—Fe1—C2'—C1'	−82.1 (2)	C38—Fe2—C34—C33	−38.6 (5)
C7—Fe1—C2'—C1'	−146.5 (13)	C35—Fe2—C34—C33	−124.7 (2)
C3—Fe1—C2'—C1'	−148.7 (13)	C39—Fe2—C34—C33	−80.1 (2)
C6—Fe1—C2'—C1'	171.2 (4)	C37—Fe2—C34—C33	152.1 (4)
C8—Fe1—C2'—C3'	164.0 (6)	C36—Fe2—C34—C33	−167.60 (19)
C1'—Fe1—C2'—C3'	119.33 (16)	C30—Fe2—C35—C36	−78.7 (2)
C5'—Fe1—C2'—C3'	81.1 (2)	C31—Fe2—C35—C36	−40.8 (4)
C9—Fe1—C2'—C3'	−155.3 (4)	C32—Fe2—C35—C36	156.8 (4)
C4—Fe1—C2'—C3'	13.5 (5)	C38—Fe2—C35—C36	80.85 (17)
C5—Fe1—C2'—C3'	57.5 (3)	C39—Fe2—C35—C36	118.8 (2)
C10—Fe1—C2'—C3'	−110.2 (5)	C34—Fe2—C35—C36	−121.54 (18)
C4'—Fe1—C2'—C3'	37.20 (16)	C37—Fe2—C35—C36	37.26 (16)
C7—Fe1—C2'—C3'	−27.1 (13)	C33—Fe2—C35—C36	−164.35 (18)
C3—Fe1—C2'—C3'	−29.4 (12)	C30—Fe2—C35—C39	162.56 (17)
C6—Fe1—C2'—C3'	−69.4 (4)	C31—Fe2—C35—C39	−159.6 (3)
C1'—C2'—C3'—C4'	0.0	C32—Fe2—C35—C39	38.1 (5)
Fe1—C2'—C3'—C4'	−58.6 (3)	C38—Fe2—C35—C39	−37.94 (14)
C1'—C2'—C3'—Fe1	58.6 (3)	C34—Fe2—C35—C39	119.68 (17)
C8—Fe1—C3'—C4'	−26.2 (13)	C37—Fe2—C35—C39	−81.52 (16)
C1'—Fe1—C3'—C4'	82.0 (2)	C33—Fe2—C35—C39	76.87 (19)
C5'—Fe1—C3'—C4'	37.67 (15)	C36—Fe2—C35—C39	−118.8 (2)
C9—Fe1—C3'—C4'	170.7 (4)	C39—C35—C36—C37	0.7 (3)
C4—Fe1—C3'—C4'	−16.7 (14)	Fe2—C35—C36—C37	−58.8 (2)
C5—Fe1—C3'—C4'	20.0 (3)	C39—C35—C36—Fe2	59.50 (18)
C10—Fe1—C3'—C4'	−151.0 (4)	C30—Fe2—C36—C35	120.25 (18)
C2'—Fe1—C3'—C4'	119.83 (18)	C31—Fe2—C36—C35	162.34 (18)
C7—Fe1—C3'—C4'	−66.7 (5)	C32—Fe2—C36—C35	−164.1 (3)
C3—Fe1—C3'—C4'	140.7 (9)	C38—Fe2—C36—C35	−82.83 (17)
C6—Fe1—C3'—C4'	−107.7 (5)	C39—Fe2—C36—C35	−38.54 (15)
C8—Fe1—C3'—C2'	−146.0 (13)	C34—Fe2—C36—C35	79.8 (2)
C1'—Fe1—C3'—C2'	−37.82 (15)	C37—Fe2—C36—C35	−119.9 (2)
C5'—Fe1—C3'—C2'	−82.2 (2)	C33—Fe2—C36—C35	49.7 (5)
C9—Fe1—C3'—C2'	50.9 (5)	C30—Fe2—C36—C37	−119.86 (18)
C4—Fe1—C3'—C2'	−136.5 (14)	C31—Fe2—C36—C37	−77.8 (2)
C5—Fe1—C3'—C2'	−99.8 (3)	C32—Fe2—C36—C37	−44.2 (4)
C10—Fe1—C3'—C2'	89.2 (5)	C38—Fe2—C36—C37	37.07 (15)
C4'—Fe1—C3'—C2'	−119.83 (18)	C35—Fe2—C36—C37	119.9 (2)
C7—Fe1—C3'—C2'	173.5 (5)	C39—Fe2—C36—C37	81.35 (17)
C3—Fe1—C3'—C2'	20.8 (8)	C34—Fe2—C36—C37	−160.29 (18)
C6—Fe1—C3'—C2'	132.4 (5)	C33—Fe2—C36—C37	169.6 (4)
C2'—C3'—C4'—C5'	0.0	C35—C36—C37—C38	0.0 (3)
Fe1—C3'—C4'—C5'	−58.4 (3)	Fe2—C36—C37—C38	−58.68 (19)
C2'—C3'—C4'—Fe1	58.4 (3)	C35—C36—C37—Fe2	58.7 (2)
C1'—C5'—C4'—C3'	0.0	C30—Fe2—C37—C38	−160.68 (18)
Fe1—C5'—C4'—C3'	59.5 (3)	C31—Fe2—C37—C38	−120.92 (19)
C1'—C5'—C4'—Fe1	−59.5 (3)	C32—Fe2—C37—C38	−79.7 (2)
C8—Fe1—C4'—C3'	172.3 (5)	C35—Fe2—C37—C38	83.10 (17)
C1'—Fe1—C4'—C3'	−81.3 (2)	C39—Fe2—C37—C38	38.33 (16)
C5'—Fe1—C4'—C3'	−119.50 (19)	C34—Fe2—C37—C38	174.1 (4)
C9—Fe1—C4'—C3'	−160.3 (9)	C33—Fe2—C37—C38	−52.8 (4)
C4—Fe1—C4'—C3'	10.2 (8)	C36—Fe2—C37—C38	120.3 (2)
C5—Fe1—C4'—C3'	−122.6 (8)	C30—Fe2—C37—C36	79.0 (2)
C10—Fe1—C4'—C3'	57.3 (5)	C31—Fe2—C37—C36	118.80 (19)
C2'—Fe1—C4'—C3'	−37.26 (16)	C32—Fe2—C37—C36	160.06 (18)
C7—Fe1—C4'—C3'	132.3 (6)	C38—Fe2—C37—C36	−120.3 (2)
C3—Fe1—C4'—C3'	−18.2 (4)	C35—Fe2—C37—C36	−37.18 (16)
C6—Fe1—C4'—C3'	90.3 (5)	C39—Fe2—C37—C36	−81.95 (17)
C8—Fe1—C4'—C5'	−68.2 (6)	C34—Fe2—C37—C36	53.8 (4)
C1'—Fe1—C4'—C5'	38.17 (16)	C33—Fe2—C37—C36	−173.1 (3)
C9—Fe1—C4'—C5'	−40.8 (10)	C36—C37—C38—C39	−0.7 (3)
C4—Fe1—C4'—C5'	129.7 (9)	Fe2—C37—C38—C39	−59.72 (18)
C5—Fe1—C4'—C5'	−3.1 (8)	C36—C37—C38—Fe2	59.04 (19)
C10—Fe1—C4'—C5'	176.8 (5)	C30—Fe2—C38—C37	48.0 (4)
C2'—Fe1—C4'—C5'	82.2 (2)	C31—Fe2—C38—C37	76.0 (2)
C7—Fe1—C4'—C5'	−108.2 (6)	C32—Fe2—C38—C37	117.0 (2)
C3—Fe1—C4'—C5'	101.3 (4)	C35—Fe2—C38—C37	−80.39 (18)
C6—Fe1—C4'—C5'	−150.2 (5)	C39—Fe2—C38—C37	−118.7 (2)
C8—Fe1—C6—C10	83.73 (17)	C34—Fe2—C38—C37	−174.4 (3)
C5'—Fe1—C6—C10	167.9 (5)	C33—Fe2—C38—C37	156.98 (19)
C9—Fe1—C6—C10	38.52 (15)	C36—Fe2—C38—C37	−37.01 (17)
C4—Fe1—C6—C10	−127.8 (4)	C30—Fe2—C38—C39	166.6 (3)
C5—Fe1—C6—C10	−168.3 (4)	C31—Fe2—C38—C39	−165.34 (17)
C2'—Fe1—C6—C10	−71.0 (6)	C32—Fe2—C38—C39	−124.31 (18)
C4'—Fe1—C6—C10	−154.8 (5)	C35—Fe2—C38—C39	38.27 (15)
C7—Fe1—C6—C10	120.5 (2)	C34—Fe2—C38—C39	−55.8 (4)
C3—Fe1—C6—C10	−84.2 (4)	C37—Fe2—C38—C39	118.7 (2)
C8—Fe1—C6—C7	−36.82 (16)	C33—Fe2—C38—C39	−84.36 (19)
C5'—Fe1—C6—C7	47.4 (5)	C36—Fe2—C38—C39	81.65 (17)
C9—Fe1—C6—C7	−82.03 (17)	C37—C38—C39—C40	168.5 (3)
C4—Fe1—C6—C7	111.7 (4)	Fe2—C38—C39—C40	108.5 (3)
C5—Fe1—C6—C7	71.1 (5)	C37—C38—C39—C35	1.1 (3)
C10—Fe1—C6—C7	−120.5 (2)	Fe2—C38—C39—C35	−58.93 (18)
C2'—Fe1—C6—C7	168.4 (6)	C37—C38—C39—Fe2	60.01 (18)
C4'—Fe1—C6—C7	84.7 (5)	C36—C35—C39—C40	−170.0 (2)
C3—Fe1—C6—C7	155.2 (4)	Fe2—C35—C39—C40	−109.9 (2)
C10—C6—C7—C8	−1.3 (3)	C36—C35—C39—C38	−1.1 (3)
Fe1—C6—C7—C8	57.1 (2)	Fe2—C35—C39—C38	59.02 (18)
C10—C6—C7—Fe1	−58.38 (19)	C36—C35—C39—Fe2	−60.10 (19)
C1'—Fe1—C7—C8	51.3 (5)	C30—Fe2—C39—C40	68.2 (4)
C5'—Fe1—C7—C8	84.7 (4)	C31—Fe2—C39—C40	−88.6 (4)
C9—Fe1—C7—C8	−39.42 (16)	C32—Fe2—C39—C40	−52.5 (2)
C4—Fe1—C7—C8	152.4 (5)	C38—Fe2—C39—C40	−126.3 (3)
C5—Fe1—C7—C8	108.2 (5)	C35—Fe2—C39—C40	114.7 (2)
C10—Fe1—C7—C8	−84.12 (17)	C34—Fe2—C39—C40	32.6 (2)
C2'—Fe1—C7—C8	−171.5 (12)	C37—Fe2—C39—C40	−164.1 (2)
C4'—Fe1—C7—C8	127.6 (5)	C33—Fe2—C39—C40	−10.5 (2)
C3—Fe1—C7—C8	−170.1 (5)	C36—Fe2—C39—C40	152.3 (2)
C6—Fe1—C7—C8	−120.8 (2)	C30—Fe2—C39—C38	−165.5 (3)
C8—Fe1—C7—C6	120.8 (2)	C31—Fe2—C39—C38	37.7 (4)
C1'—Fe1—C7—C6	172.2 (4)	C32—Fe2—C39—C38	73.8 (2)
C5'—Fe1—C7—C6	−154.4 (4)	C35—Fe2—C39—C38	−119.0 (2)
C9—Fe1—C7—C6	81.42 (17)	C34—Fe2—C39—C38	158.90 (17)
C4—Fe1—C7—C6	−86.8 (5)	C37—Fe2—C39—C38	−37.81 (16)
C5—Fe1—C7—C6	−131.0 (5)	C33—Fe2—C39—C38	115.84 (17)
C10—Fe1—C7—C6	36.72 (16)	C36—Fe2—C39—C38	−81.42 (17)
C2'—Fe1—C7—C6	−50.7 (12)	C30—Fe2—C39—C35	−46.6 (4)
C4'—Fe1—C7—C6	−111.6 (5)	C31—Fe2—C39—C35	156.7 (3)
C3—Fe1—C7—C6	−49.3 (6)	C32—Fe2—C39—C35	−167.23 (18)
C6—C7—C8—C9	2.1 (3)	C38—Fe2—C39—C35	119.0 (2)
Fe1—C7—C8—C9	60.55 (18)	C34—Fe2—C39—C35	−82.14 (19)
C6—C7—C8—Fe1	−58.4 (2)	C37—Fe2—C39—C35	81.15 (17)
C1'—Fe1—C8—C7	−152.7 (5)	C33—Fe2—C39—C35	−125.20 (17)
C5'—Fe1—C8—C7	−110.1 (5)	C36—Fe2—C39—C35	37.54 (16)
C9—Fe1—C8—C7	117.7 (2)	C58—N2—C40—C39	−169.7 (2)
C4—Fe1—C8—C7	−55.6 (6)	Pd2—N2—C40—C39	2.5 (4)
C5—Fe1—C8—C7	−90.5 (4)	C38—C39—C40—N2	21.2 (5)
C10—Fe1—C8—C7	79.50 (17)	C35—C39—C40—N2	−172.9 (2)
C2'—Fe1—C8—C7	176.7 (5)	Fe2—C39—C40—N2	106.4 (3)
C4'—Fe1—C8—C7	−71.3 (4)	C47—P2—C41—C42	18.8 (3)
C3—Fe1—C8—C7	156.9 (10)	C52—P2—C41—C42	134.4 (2)
C6—Fe1—C8—C7	36.48 (16)	Pd2—P2—C41—C42	−116.6 (2)
C1'—Fe1—C8—C9	89.6 (5)	C47—P2—C41—C46	−161.6 (2)
C5'—Fe1—C8—C9	132.2 (5)	C52—P2—C41—C46	−46.0 (3)
C4—Fe1—C8—C9	−173.3 (6)	Pd2—P2—C41—C46	63.0 (2)
C5—Fe1—C8—C9	151.9 (4)	C46—C41—C42—C43	0.4 (4)
C10—Fe1—C8—C9	−38.18 (15)	P2—C41—C42—C43	−180.0 (2)
C2'—Fe1—C8—C9	59.0 (5)	C41—C42—C43—C44	0.3 (5)
C4'—Fe1—C8—C9	171.0 (4)	C42—C43—C44—C45	−0.4 (5)
C7—Fe1—C8—C9	−117.7 (2)	C43—C44—C45—C46	−0.2 (5)
C3—Fe1—C8—C9	39.2 (11)	C42—C41—C46—C45	−1.0 (4)
C6—Fe1—C8—C9	−81.19 (17)	P2—C41—C46—C45	179.3 (2)
C7—C8—C9—C11	−166.1 (2)	C44—C45—C46—C41	1.0 (5)
Fe1—C8—C9—C11	−104.3 (2)	C52—P2—C47—C48	−14.2 (3)
C7—C8—C9—C10	−2.2 (3)	C41—P2—C47—C48	100.1 (2)
Fe1—C8—C9—C10	59.65 (17)	Pd2—P2—C47—C48	−124.7 (2)
C7—C8—C9—Fe1	−61.82 (19)	C52—P2—C47—C53	164.2 (2)
C8—Fe1—C9—C11	116.7 (3)	C41—P2—C47—C53	−81.6 (2)
C1'—Fe1—C9—C11	8.9 (6)	Pd2—P2—C47—C53	53.6 (2)
C5'—Fe1—C9—C11	50.1 (5)	C53—C47—C48—C49	0.1 (4)
C4—Fe1—C9—C11	−78.2 (11)	P2—C47—C48—C49	178.5 (2)
C5—Fe1—C9—C11	60.2 (5)	C47—C48—C49—C50	0.9 (5)
C10—Fe1—C9—C11	−124.5 (2)	C48—C49—C50—C51	−1.3 (5)
C2'—Fe1—C9—C11	−34.4 (6)	C49—C50—C51—C53	0.5 (5)
C4'—Fe1—C9—C11	83.3 (8)	C47—P2—C52—C58	−100.3 (2)
C7—Fe1—C9—C11	154.9 (2)	C41—P2—C52—C58	143.84 (19)
C3—Fe1—C9—C11	−53.3 (4)	Pd2—P2—C52—C58	22.3 (2)
C6—Fe1—C9—C11	−161.9 (2)	C47—P2—C52—C54	81.5 (3)
C8—Fe1—C9—C10	−118.7 (2)	C41—P2—C52—C54	−34.4 (3)
C1'—Fe1—C9—C10	133.5 (6)	Pd2—P2—C52—C54	−155.9 (2)
C5'—Fe1—C9—C10	174.6 (5)	C50—C51—C53—C47	0.6 (5)
C4—Fe1—C9—C10	46.3 (11)	C48—C47—C53—C51	−0.9 (4)
C5—Fe1—C9—C10	−175.2 (4)	P2—C47—C53—C51	−179.3 (2)
C2'—Fe1—C9—C10	90.1 (5)	C58—C52—C54—C55	−0.8 (4)
C4'—Fe1—C9—C10	−152.2 (8)	P2—C52—C54—C55	177.3 (2)
C7—Fe1—C9—C10	−80.58 (16)	C52—C54—C55—C56	−0.7 (5)
C3—Fe1—C9—C10	71.2 (3)	C54—C55—C56—C57	1.7 (5)
C6—Fe1—C9—C10	−37.34 (15)	C55—C56—C57—C58	−1.1 (4)
C1'—Fe1—C9—C8	−107.8 (6)	C56—C57—C58—C52	−0.4 (4)
C5'—Fe1—C9—C8	−66.6 (5)	C56—C57—C58—N2	179.3 (2)
C4—Fe1—C9—C8	165.0 (10)	C54—C52—C58—C57	1.4 (4)
C5—Fe1—C9—C8	−56.5 (5)	P2—C52—C58—C57	−177.00 (19)
C10—Fe1—C9—C8	118.7 (2)	C54—C52—C58—N2	−178.4 (2)
C2'—Fe1—C9—C8	−151.2 (5)	P2—C52—C58—N2	3.3 (3)
C4'—Fe1—C9—C8	−33.5 (9)	C40—N2—C58—C57	−37.7 (3)
C7—Fe1—C9—C8	38.13 (17)	Pd2—N2—C58—C57	148.9 (2)
C3—Fe1—C9—C8	−170.1 (3)	C40—N2—C58—C52	142.0 (2)
C6—Fe1—C9—C8	81.38 (17)	Pd2—N2—C58—C52	−31.3 (3)
C7—C6—C10—C9	−0.1 (3)		
